# Diagnostic value of metagenomic next-generation sequencing in deep neck space infections: a retrospective study of 32 patients

**DOI:** 10.3389/fcimb.2026.1874210

**Published:** 2026-07-17

**Authors:** Zheng Wang, Huiqian Yang, Jian Liu, Xiaoming Li

**Affiliations:** Department of Otolaryngology, Shandong Provincial Hospital Affiliated to Shandong First Medical University, Jinan, Shandong, China

**Keywords:** anaerobic bacteria, antibiotic therapy, clinical microbiology, deep neck space infection, metagenomic next- generation sequencing, pathogen detection, polymicrobial infection, surgical drainage

## Abstract

**Introduction:**

Deep neck space infections (DNSI) are rapidly progressive suppurative conditions in which early identification of causative pathogens is critical for clinical decision-making. This study evaluated the diagnostic performance and clinical utility of metagenomic next-generation sequencing (mNGS) in patients with DNSI.

**Methods:**

In this retrospective observational study, 32 patients with radiologically confirmed DNSI who underwent surgical drainage between October 2023 and August 2025 were included. Intraoperative purulent specimens were analyzed using both conventional bacterial culture and mNGS. A composite clinical reference standard integrating clinical presentation, imaging findings, surgical observations, inflammatory markers, and expert assessment was used to evaluate the clinical relevance of detected microorganisms.

**Results:**

mNGS detected microbial DNA in 84.4% of patients and demonstrated a broader pathogen detection spectrum and shorter reporting time than conventional culture, particularly for anaerobic and fastidious organisms. Frequently detected organisms included Prevotella spp. and Streptococcus constellatus. Interpretation of these findings required careful consideration of anatomical involvement, organism abundance, and prior antimicrobial exposure to distinguish clinically relevant pathogens from colonizing organisms or residual nonviable DNA. Discordant findings between culture and mNGS, including culture-positive/mNGS-negative and dual-negative cases, were observed and likely reflected differences in sampling adequacy, organism viability, sequencing depth, and methodological limitations. Antimicrobial therapy was adjusted in selected patients following mNGS reporting.

**Discussion:**

mNGS may serve as a valuable adjunct to conventional microbiological diagnostics by expanding pathogen detection in selected DNSI cases, particularly when fastidious or anaerobic organisms are involved. However, its results should be interpreted cautiously in the context of clinical and microbiological findings. Prospective controlled studies are needed to further define the clinical role of mNGS in the management of DNSI.

## Introduction

Deep neck space infections (DNSI) comprise a heterogeneous group of rapidly progressive suppurative conditions involving the deep cervical fascial compartments, and remain potentially life-threatening infections despite advances in imaging, antimicrobial therapy, and surgical management (Yang et al., 2015; Hansen et al., 2020). Abscesses and phlegmons in this region are broadly categorized into odontogenic and non−odontogenic forms, with the latter most commonly arising from tonsillogenic or pharyngeal sources, as demonstrated in recent epidemiological analyses of head and neck infections (Yankov and Stoev, 2023). The present study focuses specifically on non−odontogenic infections. Once established, DNSI may extend along the deep cervical fascial planes, and although descending spread into the mediastinum is well recognized, ascending propagation through the valveless facial and pterygoid venous plexuses may also occur, potentially resulting in cavernous sinus thrombosis or intracranial suppuration, complications that have been increasingly documented in contemporary cohorts (Yankov and Stoev, 2023). Retropharyngeal abscesses may additionally originate from suppurative lymphadenitis, in which the capsule of an infected lymph node undergoes lysis and purulent material dissects into the retropharyngeal space, a mechanism emphasized in recent etiological studies of lymph node–origin neck abscesses (Yankov, 2023a).

The microbial etiology of DNSI is typically polymicrobial, reflecting the intricate immunomicrobial environment of the oral cavity and upper airway, where aerobic and anaerobic organisms coexist within biofilms, interact synergistically under hypoxic conditions, and exploit disruptions in mucosal integrity or host immune function. Conventional bacterial culture remains widely used in clinical practice; however, its diagnostic yield is limited by the oxygen sensitivity of anaerobes, the fastidious nature of certain organisms, and the frequent administration of empirical antibiotics before specimen collection, all of which may reduce the likelihood of recovering viable pathogens (Hansen et al., 2020; Reissier et al., 2023; Yankov, 2023). Moreover, culture−based methods require prolonged incubation and may fail to distinguish between true pathogens and background flora when mixed growth occurs, complicating interpretation in infections that originate from microbially dense anatomical regions.

Metagenomic next−generation sequencing (mNGS) has emerged as a culture−independent approach capable of detecting microbial DNA from a broad range of organisms, including bacteria, fungi, and viruses, without requiring microbial viability. This methodological advantage allows mNGS to identify fastidious or anaerobic organisms that may be missed by culture; however, it also introduces interpretive challenges, as mNGS detects nucleic acids irrespective of organism viability and may identify commensal taxa, colonizers, or residual DNA from organisms suppressed by prior antibiotic exposure (Kalbitz et al., 2024; Zhang et al., 2024). In DNSI, where oral anaerobes and commensal organisms are frequently present in non−invasive states, distinguishing clinically relevant pathogens from background microbial signals requires careful integration of sequencing data with clinical presentation, imaging findings, anatomical involvement, inflammatory markers, and surgical observations. Recent studies have highlighted the potential of mNGS to broaden pathogen detection in head and neck infections, but also emphasize the need for cautious interpretation in polymicrobial environments (Gu et al., 2019; Li et al., 2023; Zheng et al., 2023).

Although mNGS has shown promise in expanding the detectable microbial spectrum in various infectious diseases, its clinical value in DNSI—particularly its role in guiding antimicrobial decision−making—remains incompletely defined. Existing studies are limited by small sample sizes, heterogeneous methodologies, and the absence of standardized interpretive frameworks for determining microbial relevance in polymicrobial infections (Kalbitz et al., 2024; Lei et al., 2025) Furthermore, the retrospective nature of most available data precludes establishing causal relationships between mNGS−informed antimicrobial adjustments and clinical outcomes, especially in a condition where surgical drainage, empirical broad−spectrum antibiotics, and supportive care constitute the primary determinants of recovery. Against this background, the present retrospective study evaluates the diagnostic contribution of mNGS in patients with DNSI who underwent surgical drainage, comparing its detection spectrum, turnaround time, and concordance with conventional culture, while critically examining the interpretive challenges posed by polymicrobial detection, prior antibiotic exposure, and the presence of oral commensals. Rather than assessing superiority, the study aims to clarify how mNGS may complement existing diagnostic approaches and to identify the methodological and clinical considerations necessary for its appropriate interpretation in the management of deep neck space infections.

## Materials and methods

### Study design and patients

This retrospective observational study included patients diagnosed with deep neck space infections (DNSI) who were admitted to the Department of Otolaryngology–Head and Neck Surgery at Shandong Provincial Hospital Affiliated to Shandong First Medical University between October 2023 and August 2025. DNSI was defined according to previously described diagnostic criteria involving abscesses or phlegmonous changes within one or more deep cervical fascial spaces, including the retropharyngeal, parapharyngeal, submandibular, carotid sheath, and contiguous compartments (Yang et al., 2015; Hansen et al., 2020). A total of 41 patients were initially screened based on clinical presentation and imaging; 9 were excluded due to insufficient imaging, absence of purulent material during surgery, or incomplete clinical records, leaving 32 patients who met all eligibility criteria.

Both male and female patients were eligible without sex-based restrictions, and no predefined upper age limit was applied. All patients underwent contrast-enhanced CT or MRI on admission to evaluate the extent of infection. Empirical broad-spectrum antibiotics were initiated immediately according to standard clinical practice for severe DNSI, and surgical drainage was performed via intraoral or external cervical approaches depending on abscess location.

### Patient screening and case selection

Patients were included if they met the following criteria:

1. radiologically confirmed deep neck infection involving deep cervical fascial spaces;
2. intraoperative acquisition of purulent material suitable for microbiological testing;
3. availability of complete clinical, laboratory, and imaging data.

Exclusion criteria included:

1. insufficient imaging to localize the infection to deep neck spaces;
2. absence of purulent material during surgery;
3. incomplete clinical records.
4. These criteria ensured that only patients with true deep neck space infections were included, consistent with prior studies.

### Specimen collection and handling

During surgical drainage, purulent material was collected using sterile syringes and immediately divided into two aliquots: one for conventional bacterial culture and one for metagenomic next-generation sequencing (mNGS). To minimize contamination, specimens were obtained from the deepest accessible portion of the abscess cavity after removal of superficial debris. All samples were transported under controlled conditions of collection.

### Conventional culture

Specimens for culture were processed in the hospital microbiology laboratory. Aerobic and anaerobic cultures were performed using standard media, and when indicated, blood culture bottles were incubated in an automated blood culture system. Bacterial isolates were identified using the VITEK 2 Compact automated identification system, and antimicrobial susceptibility testing was performed according to CLSI guidelines. The limitations of anaerobic culture—particularly the oxygen sensitivity of anaerobes during transport and processing—were acknowledged as potential contributors to reduced culture yield (Yankov, 2023).

### mNGS procedure

Purulent specimens designated for metagenomic next-generation sequencing (mNGS) were transported to Beijing CapitalBio Medical Laboratory, where nucleic acid extraction and library preparation were performed using the MAPMI Sample Preparation and Nucleic Acid Extraction Kit (CapitalBio Technology, Beijing, China). For samples containing cellular components, host cells underwent differential lysis followed by nuclease digestion to reduce human nucleic acid background, whereas plasma-like specimens without intact host cells were processed directly to maximize recovery of microbial cell-free DNA. Library preparation followed the MAPMI protocol, including enzymatic fragmentation (for cellular DNA), end-repair and A-tailing, adapter ligation, and PCR amplification. Library quality and concentration were assessed using the Qubit dsDNA HS Assay Kit (Thermo Fisher Scientific, USA). Sequencing was performed on the CBTSeq2 Pro platform (CapitalBio Technology, Chengdu, China) using a dual-index strategy, generating ≥20 million single-end reads (75 bp) per sample to ensure adequate depth for pathogen detection. Each sequencing batch included positive controls containing known microorganisms and negative controls consisting of human genomic DNA to monitor background contamination.

### Bioinformatic processing and contamination control

Bioinformatic analysis was performed using MAPMI v3.7, an automated pipeline for pathogen detection. The workflow consisted of the following steps:

1. Quality control: Adapter trimming, removal of low-complexity and repetitive reads, and truncation of reads with Phred quality < Q20 were performed using fastp (v0.20.0).
2. Host-read depletion: High-quality reads ≥50 bp were aligned to the human telomere-to-telomere reference genome HG0002 (v1.1) using Bowtie2 (v2.3.4.1). Reads mapping to the human genome were removed.
3. Microbial alignment: Non-human reads were aligned using Bowtie2 to the MAPMI reference database (v4.1), which integrates multiple curated microbial resources, including:
NCBI nt database
NCBI RefSeq database
NCBI GenBank database
FDA-ARGOS
FDA Reference Viral Database
World Data Center for Microorganisms (WDCM)
BV-BRC, PATRIC, EuPathDB, and other pathogen-specific repositories.
This database includes bacteria, fungi, viruses, and parasites with standardized taxonomic annotation.
4. Taxonomic classification: Reads were assigned to microbial species based on alignment quality, unique read count, and genome coverage.
5. Contamination filtering: A laboratory “blacklist” was generated from negative controls processed over the preceding 90 days. Organisms detected in negative controls were excluded unless the patient sample exhibited a read count at least 10-fold higher than the control.
6. Reporting thresholds: Organisms were reported when they met the following quantitative criteria:
Bacteria/fungi: ≥3 unique reads and ≥1% genome coverage
Mycobacterium tuberculosis complex: ≥1 unique read
Viruses: ≥10 reads
Relative abundance ≥0.01% after host-read removal.
These thresholds were consistent with the laboratory’s validated detection standards and were based on the laboratory’s validated criteria and internal quality control data.
7. Output and interpretation: The final output included taxonomic identity, unique read count, reads per million (RPM), relative abundance, genome coverage, and pathogenicity annotation. Genome coverage plots were generated for manual review. Clinical relevance was determined using the composite clinical reference standard described in the Methods section.

### Interpretation of microbiological findings

Because no universally accepted microbiological gold standard exists for DNSI, a composite clinical reference standard was used to interpret microbial relevance. This assessment was performed by two senior otolaryngologists and one infectious disease specialist, who jointly reviewed each case. The evaluation was not blinded to culture or mNGS results, as clinical interpretation required integration of all available information. Disagreements were resolved through consensus discussion.

An organism was considered clinically relevant only when it met all of the following criteria:

1. Anatomical plausibility, based on imaging and intraoperative findings.
2. Correlation with inflammatory markers and clinical severity.
3. Sufficient sequencing signal, defined by read count, relative abundance, and genome coverage above laboratory thresholds.
4. Consistency with known pathogenicity in deep neck infections.
5. Exclusion of contamination, based on negative controls and environmental background profiles.

This composite approach aligns with recommendations for interpreting mNGS results in polymicrobial infections where colonization and contamination are common.

### Antibiotic exposure and antimicrobial adjustment

Empirical antibiotics were administered before specimen collection in most patients, consistent with standard DNSI management. The influence of prior antibiotic exposure on culture negativity and mNGS positivity was considered during interpretation. Antimicrobial regimens were reviewed after mNGS reporting, and adjustments were made only when sequencing identified organisms judged clinically relevant and not covered by empirical therapy.

### Statistical analysis

Statistical analyses were performed using IBM SPSS Statistics version 26.0 (Windows 10), which has been widely used in clinical research since its release in 2019. Continuous variables were expressed as mean ± standard deviation, and categorical variables were compared using the chi-square test. A p-value < 0.05 was considered statistically significant. Subgroup analyses involving diabetic and overweight patients were exploratory in nature and limited by small sample sizes.

## Results

### Patient characteristics

A total of 32 patients met the inclusion criteria after screening 41 individuals with suspected deep neck infection, and their demographic and clinical characteristics are summarized in **Table 1**. All patients had radiologically confirmed involvement of one or more deep cervical fascial spaces, consistent with previously described anatomical patterns of DNSI (Yankov, 2023a; Yankov and Stoev, 2023; Yankov and Stoev, 2023). Fever was present in 22 patients on admission, and common symptoms included dysphagia, dyspnea, cervical pain, and localized swelling. Laboratory tests demonstrated elevated WBC, neutrophils, and CRP in most patients, reflecting acute inflammatory responses. Inflammatory biomarkers have been reported to correlate with disease severity and purulent inflammatory activity in deep neck infections (Yankov, 2023b). Twelve patients had diabetes mellitus and 12 were classified as overweight (BMI > 24), comorbidities known to influence host immune function and oral microbial composition (Negrini et al., 2021; Schamarek et al., 2023; Kong et al., 2024).The clinical characteristics are summarized in **Table 1**.

**Table 1:** Patient characteristics.

n=32	
Age (mean ± SD)	47.9 ± 20.0
Gender	
Male	20
Female	12
Clinical Diagnosis	
Cervical Space Infection	32
Mediastinal Infection	3
Congenital Branchial Cleft Fistula	1
Cervical Mass with Infection	1
Comorbidities	
Diabetes Mellitus (DM)	12
Hypertension (HTN)	3
Alcoholic Cirrhosis (AC)	1
Hepatitis B Virus (HBV)	1
Esophageal Perforation	1
History of Heart Failure (HF)	1
Rheumatoid Arthritis (RA)	1
Guillain-Barré Syndrome (GBS)	1
Blood Test Results (mean ± SD)	
WBC(10^9/L)	10.5 ± 3.7
NE(10^9/L)	8.2 ± 3.6
CRP(mg/L)	107.6 ± 95.2
Clinical Outcomes	
Clinical Improvement	29
Transfer to Another Hospital	2
In-Hospital Treatment	1

The table provides detailed information on infection sites, comorbidities, prior antibiotic exposure, culture findings, and mNGS-detected organisms with corresponding relative abundance. It also outlines antimicrobial modifications when they occurred and the associated clinical context. As shown in **Table 2**, most patients presented with parapharyngeal, retropharyngeal, or deep neck infections, and diabetes mellitus was the most frequent comorbidity. Conventional culture yielded limited pathogen recovery, whereas mNGS identified a broad spectrum of aerobic and anaerobic organisms across cases. Antimicrobial regimens were adjusted in selected patients when mNGS results were considered clinically relevant in conjunction with clinical assessment, while the majority of patients demonstrated clinical improvement following surgical drainage, empirical therapy, and supportive management.

**Table 2:** Summarizes the patient-level clinical, microbiological, and treatment characteristics of all 32 individuals included in this study. **Table 2** is provided as **Supplementary Table 1**.

Patient ID	Infection site	Comorbidities	Prior antibiotic exposure	Culture result	mNGS result	Relative abundance	Antibiotic modification	Reason for modification	Clinical outcome
1	Mediastinal infection	Bronchitis	Cefoperazone Sodium and Sulbactam Sodium for Injection	Gram-positive cocci	Micromonas micros,Streptococcus constellatus,Fusobacterium nucleatum,Dialister pneumosintes	40.14%,6.90%,2.24%,5.92%	None	Not applicable	Clinical Improvement
2	Parapharyngeal abscess	Hypertension	Cefuroxime Sodium for Injection	No bacteria or fungi were detected	Micromonas micros	30.86%	Cefoperazone Sodium and Sulbactam Sodium for Injection	mNGS organism (Micromonas micros) considered clinically relevant	Clinical Improvement
3	Parapharyngeal + mediastinal infection	Diabetes Mellitus	Amphotericin B	Gram-positive cocci, Gram-negative bacilli	Not detected	None	Vancomycin Hydrochloride for Injection	Empirical escalation due to clinical deterioration	Transfer to Another Hospital
4	Retropharyngeal abscess	Diabetes Mellitus	Piperacillin Sodium and Tazobactam Sodium for Injection	Gram-positive cocci, Gram-negative bacilli, Proteus bacilli	Fusobacterium necrophorum,Prevotella baroniae,Streptococcus constellatus	10.44%,69.31%,1.12%	None	Not applicable	Clinical Improvement
5	Retropharyngeal + parapharyngeal infection	Diabetes Mellitus	Cefoperazone Sodium and Sulbactam Sodium for Injection+Levornidazole and Sodium Chloride Injection	Gram-positive cocci and bacilli	Streptococcus pyogenes	99.77%	None	Not applicable	Clinical Improvement
6	Right lateral pharyngeal + mediastinal infection	None	Piperacillin Sodium and Tazobactam Sodium for Injection	No bacteria or fungi were detected	Porphyromonas endodontalis,Prevotella baroniae,Slackia exigua	20.89%,9.86%,11.38%	None	Not applicable	Clinical Improvement
7	Parapharyngeal infection	Diabetes Mellitus ,Alcoholic Cirrhosis	Cefoperazone Sodium and Sulbactam Sodium for Injection+Metronidazole and Sodium Chloride Injection	Gram-negative bacillus	Klebsiella pneumoniae,Enterobacter cancerogenus	60.06%,0.47%	Imipenem and Cilastatin Sodium for Injection	mNGS detected K. pneumoniae as dominant organism	Clinical Improvement
8	Parapharyngeal infection	Diabetes Mellitus ,Hepatitis B Virus	Cefoperazone Sodium and Sulbactam Sodium for Injection	No bacteria or fungi were detected	Sneathia amnii,Peptostreptococcus anaerobius,Klebsiella pneumoniae,Prevotella denticola,Hepatitis B virus	0.02%,6.25%,0.35%,27.86%,28.65%	Cefoperazone Sodium and Sulbactam Sodium for Injection+Metronidazole and Sodium Chloride Injection	Polymicrobial anaerobic profile (Prevotella, Peptostreptococcus, Sneathia) detected by mNGS	Transfer to Another Hospital
9	Parapharyngeal + submandibular infection	None	Cefoperazone Sodium and Sulbactam Sodium for Injection+Levornidazole and Sodium Chloride Injection+Metronidazole and Sodium Chloride Injection	No bacteria or fungi were detected	Micromonas micros	34.39%	None	Not applicable	Clinical Improvement
10	Parapharyngeal infection	Diabetes Mellitus ,Hypertension	Piperacillin Sodium and Tazobactam Sodium for Injection	Gram-positive cocci, Gram-negative bacilli	Micromonas micros,Streptococcus anginosus,Prevotella intermedia	17.43%,0.36%,1.77%	Imipenem and Cilastatin Sodium for Injection	mNGS organisms (M. micros, S. anginosus, P. intermedia) considered clinically relevant	Clinical Improvement
11	Soft-tissue infection + branchial cleft fistula	None	Fluconazole and Sodium Chloride Injection+Cefoperazone Sodium and Sulbactam Sodium for Injection+Ornidazole and Sodium Chloride Injection	No bacteria or fungi were detected	Saccharomyces cerevisiae	2.96%	None	Not applicable	Clinical Improvement
12	Parapharyngeal infection	None	Cefoperazone Sodium and Sulbactam Sodium for Injection	No bacteria or fungi were detected	Not detected	None	None	Not applicable	Clinical Improvement
13	Retropharyngeal abscess	Pyriform sinus fistula	Piperacillin Sodium and Tazobactam Sodium for Injection	Gram-positive cocci	Micromonas micros,Prevotella oralis,Streptococcus constellatus,Fusobacterium nucleatum,Slackia exigua,Dialister pneumosintes	28.15%,5.65%,0.54%,0.96%,1.83%,0.21%	None	Not applicable	Clinical Improvement
14	Retropharyngeal infection	Esophageal Perforation	Piperacillin Sodium and Tazobactam Sodium for Injection	Gram-positive cocci, Gram-negative bacilli	Streptococcus constellatus,Fusobacterium nucleatum,Micromonas micros	0.68%,0.84%,1.09%	Piperacillin Sodium and Tazobactam Sodium for Injection+Ornidazole and Sodium Chloride Injection	Anaerobic organisms detected by mNGS	Clinical Improvement
15	Left pharyngeal abscess	History of Heart Failure	Piperacillin Sodium and Tazobactam Sodium for Injection	Gram-positive cocci, Gram-negative bacilli	Prevotella oralis,Micromonas micros,Slackia exigua,Pseudomonas aeruginosa	15.58%,14.06%,6.37%,0.02%	None	Not applicable	Clinical Improvement
16	Deep neck infection	None	Piperacillin Sodium and Tazobactam Sodium for Injection	No bacteria or fungi were detected	Not detected	None	None	Not applicable	Clinical Improvement
17	Carotid space infection	None	Cefoperazone Sodium and Sulbactam Sodium for Injection+Metronidazole and Sodium Chloride Injection	No bacteria or fungi were detected	Not detected	None	None	Not applicable	Clinical Improvement
18	Parapharyngeal infection	None	Cefoperazone Sodium and Sulbactam Sodium for Injection+Levornidazole and Sodium Chloride Injection	No bacteria or fungi were detected	Mycobacterium tuberculosis complex	93.47%	Anti-tuberculosis treatment	Mycobacterium tuberculosis complex detected by mNGS	Clinical Improvement
19	Submandibular infection	Hypertension ,Rheumatoid Arthritis	Cefoperazone Sodium and Sulbactam Sodium for Injection+Levornidazole and Sodium Chloride Injection	No bacteria or fungi were detected	Not detected	None	None	Not applicable	Clinical Improvement
20	Retropharyngeal infection	Diabetes Mellitus	Cefoperazone Sodium and Sulbactam Sodium for Injection+Metronidazole and Sodium Chloride Injection	Gram-positive cocci	Streptococcus constellatus,Corynebacterium striatum,Acinetobacter baumannii	26.33%,0.40%,2.76%	Ceftriaxone Sodium for Injection	mNGS organisms (S. constellatus, C. striatum, A. baumannii) considered clinically relevant	Clinical Improvement
21	Deep neck infection	None	Cefoperazone Sodium and Sulbactam Sodium for Injection	Gram-negative bacillus	Streptococcus pyogenes	30.07%	None	Not applicable	Clinical Improvement
22	Parapharyngeal infection	Hypertension	Cefoperazone Sodium and Sulbactam Sodium for Injection	No bacteria or fungi were detected	Tetragenococcus anaerobius,Micromonas micros,Slackia exigua,Streptococcus constellatus,Bacteroides heparinolyticus	14.60%,12.39%,1.08%,1.51%,6.00%	None	Not applicable	Clinical Improvement
23	Retropharyngeal + parapharyngeal infection	None	Cefoperazone Sodium and Sulbactam Sodium for Injection	No bacteria or fungi were detected	Streptococcus constellatus,Micromonas micros	41.77%,34.76%	Ceftriaxone Sodium for Injection	mNGS organisms (S. constellatus, M. micros) considered clinically relevant	Clinical Improvement
24	Deep infection of the left sternocleidomastoid muscle	None	Piperacillin Sodium and Tazobactam Sodium for Injection	Gram-positive cocci	Micromonas micros,Prevotella oralis,Streptococcus constellatus,Porphyromonas endodontalis,Slackia exigua	35.86%,9.1%,0.96%,2.26%,2.37%	Vancomycin Hydrochloride for Injection+Ornidazole and Sodium Chloride Injection	Anaerobic organisms detected by mNGS	Clinical Improvement
25	Submandibular infection	None	Cefoperazone Sodium and Sulbactam Sodium for Injection+Metronidazole and Sodium Chloride Injection	Gram-positive cocci, Gram-negative bacilli	Fusobacterium nucleatum,Slackia exigua,Streptococcus anginosus,Prevotella oralis	1.48%,1.53%,0.70%,47.50%	None	Not applicable	Clinical Improvement
26	Parapharyngeal infection	None	Meropenem for Injection	Gram-positive cocci, Gram-negative bacilli	Fusobacterium nucleatum,Segatella oris,Streptococcus constellatus	3.43%,30.25%,6.705	Ceftriaxone Sodium for Injection+Levornidazole and Sodium Chloride Injection	mNGS organisms (F. nucleatum, S. oris, S. constellatus) considered clinically relevant	Clinical Improvement
27	Retropharyngeal infection	Diabetes Mellitus ,Guillain-Barré Syndrome	Cefoperazone Sodium and Sulbactam Sodium for Injection	Gram-positive cocci	Klebsiella pneumoniae	99.54%	None	Not applicable	Clinical Improvement
28	Neck soft-tissue infection	None	Ceftriaxone Sodium for Injection	Gram-positive cocci, Gram-negative bacilli	Prevotella oralis,Streptococcus constellatus	80.87%,2.98%	Piperacillin Sodium and Tazobactam Sodium for Injection	mNGS organisms (P. oralis, S. constellatus) considered clinically relevant	Clinical Improvement
29	Parapharyngeal infection	Diabetes Mellitus	Cefoperazone Sodium and Sulbactam Sodium for Injection+Levornidazole and Sodium Chloride Injection	No bacteria or fungi were detected	Micromonas micros,Streptococcus constellatus,Bacteroides heparinolyticus	25.21%,4.44%,2.62%	Ceftriaxone Sodium for Injection	mNGS organisms (M. micros, S. constellatus, B. heparinolyticus) considered clinically relevant	Clinical Improvement
30	Thyroid-adjacent abscess	Diabetes Mellitus	Cefoperazone Sodium and Sulbactam Sodium for Injection+Levornidazole and Sodium Chloride Injection	Gram-negative bacillus	Micromonas micros,Slackia exigua	4.19%,21.54%	None	Not applicable	Clinical Improvement
31	Parapharyngeal infection	Diabetes Mellitus	Cefoperazone Sodium and Sulbactam Sodium for Injection	Gram-positive cocci, Gram-negative bacilli	Micromonas micros,Prevotella oralis,Streptococcus constellatus	6.19%,38.50%,12.75%	Levornidazole and Sodium Chloride Injection	Anaerobic organisms (P. oralis, S. constellatus) detected by mNGS	In-Hospital Treatment
32	Retropharyngeal infection	Diabetes Mellitus	Cefoperazone Sodium and Sulbactam Sodium for Injection	Gram-positive cocci	Streptococcus constellatus	90.11%	Metronidazole and Sodium Chloride Injection	mNGS organism (S. constellatus) considered clinically relevant	Clinical Improvement

### Imaging findings

**Figure 1:**
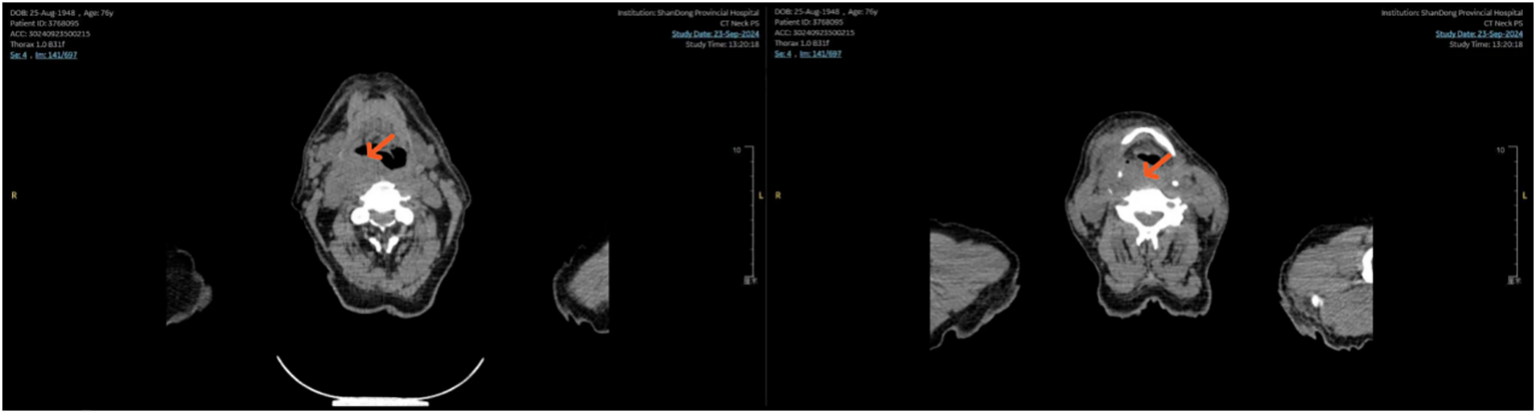
Representative CT scan of Patient 1 with deep neck space infection. Axial contrast-enhanced CT imaging demonstrates soft-tissue swelling and heterogeneous low-density areas involving the retropharyngeal space, right parapharyngeal space, and submandibular region. The irregular contours and fluid-attenuation areas are consistent with abscess formation within multiple deep cervical compartments.

**Figure 2:**
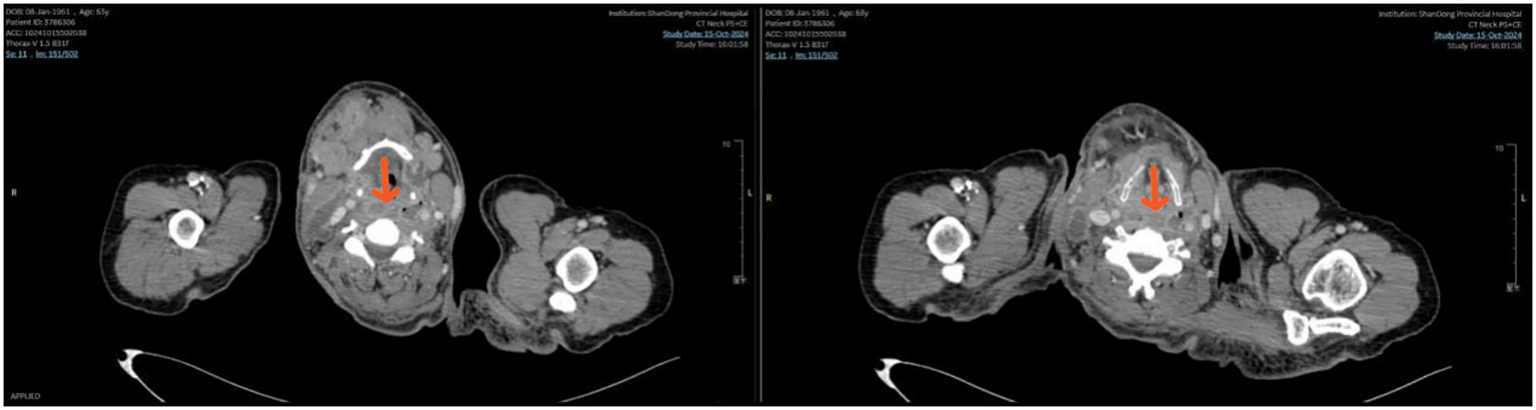
CT scan of patient 2 showing extensive bilateral cervical involvement. Contrast-enhanced CT reveals diffuse soft-tissue swelling of the bilateral cervical and pharyngeal walls, with multiple irregular low-density lesions in the right submandibular region, bilateral cervical spaces, posterior pharyngeal wall, and supraclavicular areas. These findings indicate multiloculated abscesses and widespread deep neck space infection.

Contrast-enhanced CT was performed in all patients to delineate the extent of infection. Imaging patterns were consistent with those reported in prior DNSI cohorts, including retropharyngeal, parapharyngeal, submandibular, and carotid sheath involvement. Three patients exhibited descending spread into the upper mediastinum, while others demonstrated multiloculated abscesses or phlegmonous changes. Representative imaging findings are shown in **Figures 1** and **2**.

### Pathogen identification by conventional culture and mNGS

**Figure 3:**
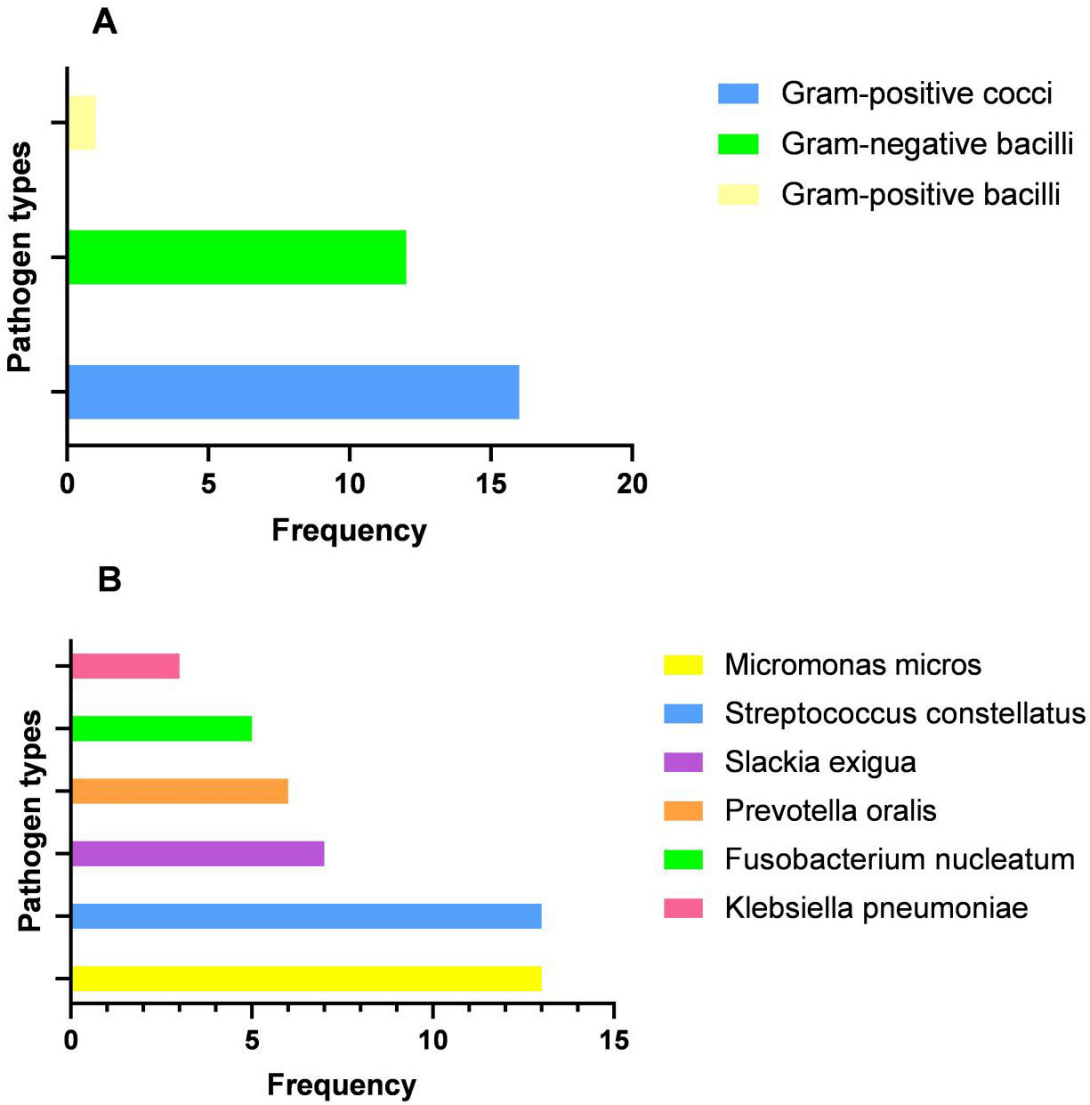
Comparison of microorganisms detected by conventional culture and mNGS. **(A)** Traditional bacterial culture identified three major categories of organisms: Gram-positive cocci, Gram-negative bacilli, and Gram-negative cocci. **(B)** Metagenomic next-generation sequencing (mNGS) detected 26 distinct microbial species, including anaerobes, fastidious bacteria, fungi, and viruses. For clarity, only the six bacterial species with the highest detection frequency are displayed.

All 32 patients underwent both conventional culture and mNGS on the same intraoperative purulent specimen. Conventional culture yielded positive results in 19 cases (59.4%), identifying primarily Gram-positive cocci, Gram-negative bacilli, and Gram-negative cocci (**Figure 3A**). In contrast, mNGS detected microbial DNA in 27 cases (84.4%), 26 microbial species were detected, including 9 Gram-positive bacteria, 14 Gram-negative bacteria, 1 virus, 1 fungus and Mycobacterium tuberculosis complex. (**Figure 3B**). These findings are consistent with previous studies demonstrating the broader pathogen detection spectrum and higher positivity rate of mNGS in clinical infectious disease diagnostics (Zheng et al., 2023; Kalbitz et al., 2024; Zhang et al., 2024; Lei et al., 2025; Sun et al., 2025).

Notably, mNGS identified several fastidious or anaerobic organisms—including Prevotella melaninogenica, Fusobacterium necrophorum, and Micromonas micros—that were not recovered by culture, reflecting the known limitations of anaerobic culture due to oxygen sensitivity and transport-related viability loss (Yankov, 2023). mNGS also detected Mycobacterium tuberculosis complex and Saccharomyces cerevisiae in isolated cases, organisms that may be difficult to culture under routine laboratory conditions.

However, many organisms detected by mNGS, such as Prevotella spp. and Streptococcus constellatus, are common oral commensals, and their detection required careful clinical correlation to determine pathogenic relevance, particularly in the context of polymicrobial environments and prior antibiotic exposure (Hao et al., 2024).

### Concordance and discordance between mNGS and culture

**Figure 4:**
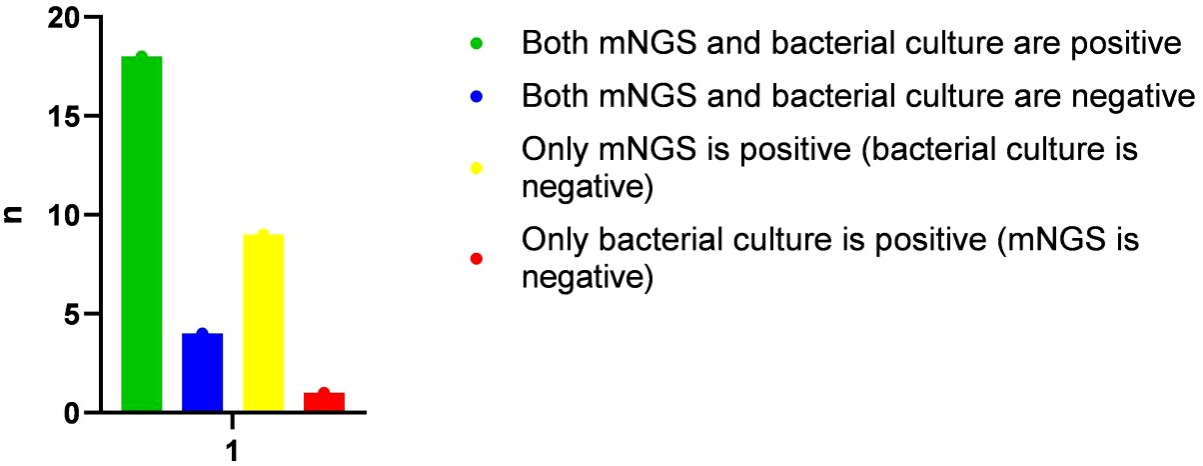
Concordance between mNGS and conventional bacterial culture in pathogen detection. This bar chart illustrates the distribution of diagnostic concordance patterns between metagenomic next-generation sequencing (mNGS) and conventional culture in 32 patients with deep neck space infections. Both methods were positive in 18 patients, both were negative in 4 patients, mNGS alone was positive in 9 patients, and culture alone was positive in 1 patient. These patterns highlight the complementary nature of the two diagnostic approaches and reflect differences in organism viability, anaerobic recovery, sequencing depth, and prior antibiotic exposure.

The concordance analysis between the two diagnostic modalities is shown in **Figure 4**. Four patients (12.5%) were negative by both methods, a finding that may reflect low bacterial load, inadequate sampling, or suppression of viable organisms by empirical antibiotics administered before specimen collection. Nine patients (28.1%) were positive only by mNGS, consistent with the ability of sequencing to detect DNA from anaerobes, fastidious organisms, or nonviable bacteria. One patient (3.1%) was positive only by culture, a discordance that may be attributed to low sequencing depth, DNA degradation, or the presence of organisms with limited genomic representation in reference databases.

These discordant patterns underscore the complementary nature of the two diagnostic approaches and highlight the need for a composite clinical reference standard when interpreting microbiological findings in DNSI.

### Turnaround time

The mean turnaround time for mNGS was approximately 1 day, whereas conventional culture required an average of 5.03 ± 0.63 days (Hao et al., 2024; Kalbitz et al., 2024; Lei et al., 2025). This difference reflects the inherent delays associated with microbial growth and incubation in culture-based methods, particularly for anaerobic or slow-growing organisms.

### Antimicrobial therapy and clinical outcomes

Antimicrobial regimens were adjusted in 15 patients after mNGS reporting. Adjustments were made only when mNGS identified anaerobic organisms not adequately covered by the empirical regimen, such as Prevotella spp. or Fusobacterium spp., or when sequencing suggested broader anaerobic involvement. Streptococcus anginosus alone did not prompt metronidazole initiation; changes were based on co-detection of anaerobes, clinical suspicion of anaerobic infection, and gaps in empirical coverage. These decisions were made in conjunction with surgical findings and overall clinical assessment.

**Figure 5:**
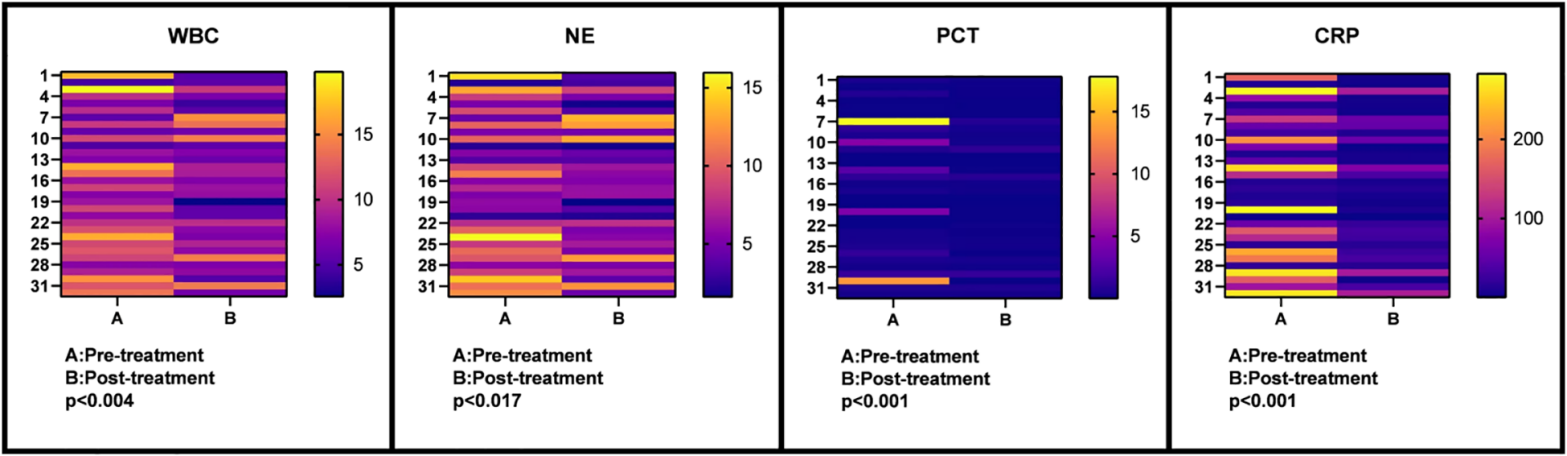
Changes in inflammatory markers before and after clinical management. Trends in white blood cell count (WBC), neutrophil percentage (N%), C−reactive protein (CRP), and procalcitonin (PCT) are shown for patients who underwent surgical drainage and subsequent antimicrobial management. All inflammatory markers demonstrated a downward trend following treatment, consistent with overall clinical improvement. These changes reflect the combined effects of surgical source control, empirical broad−spectrum antibiotics, and supportive care, rather than the isolated impact of mNGS−guided antimicrobial adjustment.

Inflammatory markers (WBC\10^9/L, Neutrophils\10^9/L, CRP\mg/L, PCT\ng/ml) declined following treatment (**Figure 5**). However, given the retrospective design and the concurrent effects of surgical drainage, empirical antibiotics, and supportive care, these improvements cannot be attributed specifically to mNGS-associated antimicrobial adjustments (Li et al., 2023).

Of the 32 patients, 29 showed clinical improvement and were discharged, 2 left the hospital voluntarily, and 1 remained hospitalized at the time of data collection.

### Subgroup analysis: diabetic and overweight patients

**Figure 6:**
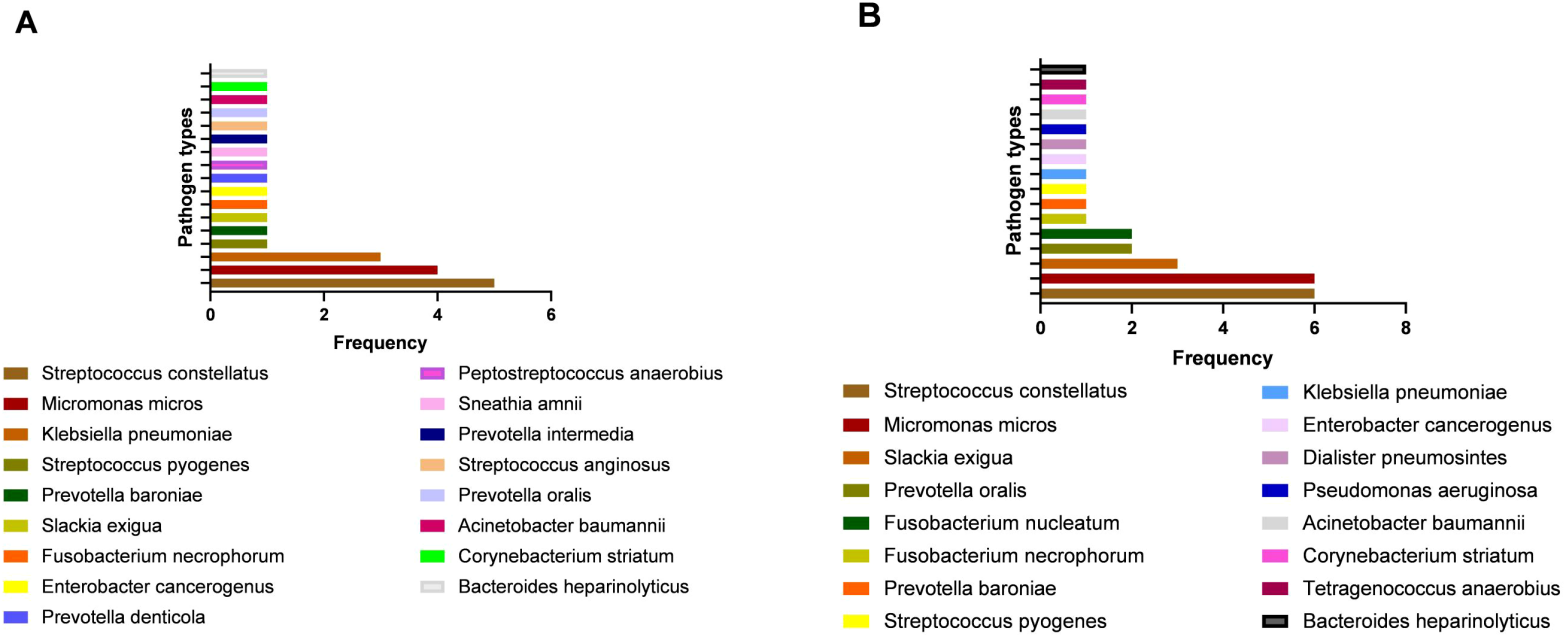
Distribution of bacterial pathogens detected by mNGS in diabetic and overweight patients. **(A)** In diabetic patients, Streptococcus constellatus was the most frequently detected organisms, along with several anaerobic and facultative anaerobic species. **(B)** In overweight patients (BMI > 24), (S) constellatus was also predominant, with a more balanced distribution of additional pathogens. mNGS demonstrated a high positivity rate and readily identified polymicrobial signatures in both subgroups.

Among the 12 diabetic and 12 overweight patients, mNGS identified Streptococcus constellatus as the most frequently detected organisms (**Figures 6A, B**), consistent with prior studies linking metabolic dysfunction to altered oral microbiota and increased susceptibility to polymicrobial infections (Negrini et al., 2021; Schamarek et al., 2023; Kong et al., 2024). mNGS demonstrated a high positivity rate and readily detected polymicrobial signatures in both subgroups.

However, these subgroup analyses were exploratory and limited by small sample sizes, and therefore should be interpreted with caution.

## Discussion

The increasing clinical application of mNGS has shifted infectious disease diagnostics from culture-dependent identification toward sequence-based pathogen profiling, particularly in culture-negative infections (Kalbitz et al., 2024; Zhang et al., 2024).Deep neck space infections (DNSI) remain critical emergencies in otolaryngology–head and neck surgery due to their rapid progression, anatomical complexity, and the potential for life-threatening complications when diagnosis or intervention is delayed. The present study evaluated the diagnostic contribution of metagenomic next-generation sequencing (mNGS) in patients with DNSI who underwent surgical drainage, with particular attention to the interpretive challenges posed by polymicrobial detection, prior antibiotic exposure, and the presence of oral commensals. Rather than assessing superiority, the aim was to clarify how mNGS may complement conventional culture and to identify methodological considerations necessary for its appropriate clinical interpretation.

Consistent with previous epidemiological analyses, DNSI in this cohort involved both descending and ascending pathways of spread, the latter of which may result in complications such as cavernous sinus thrombosis or intracranial suppuration, although these events are less frequently encountered than mediastinitis (Yankov and Stoev, 2023). The etiological diversity of DNSI reflects the complex immunomicrobial environment of the upper aerodigestive tract, where aerobic and anaerobic organisms coexist within biofilms, interact synergistically under hypoxic conditions, and exploit disruptions in mucosal integrity or host immune function. Retropharyngeal abscesses may additionally arise from suppurative lymphadenitis, in which the capsule of an infected lymph node undergoes lysis and purulent material dissects into the retropharyngeal space, a mechanism described in recent etiological studies (Yankov, 2023a). These biological features underscore the importance of diagnostic approaches capable of capturing the polymicrobial nature of DNSI while allowing clinicians to distinguish colonization from true invasive infection.

In this study, mNGS detected microbial DNA in a greater proportion of patients than conventional culture and identified a broader range of organisms, including anaerobes and fastidious species that are often difficult to recover using culture-based methods. These findings align with prior reports describing an expanded detection spectrum of mNGS in head and neck infections (Gu et al., 2019; Li et al., 2023; Zheng et al., 2023). However, broader detection does not inherently imply greater diagnostic accuracy or clinical utility, particularly in anatomical regions with dense commensal flora. Many organisms identified by mNGS—such as Prevotella spp. and Streptococcus constellatus—are common oral commensals that may be present in non-invasive states, and their detection requires careful clinical correlation to determine pathogenic relevance (Negrini et al., 2021; Reissier et al., 2023; Schamarek et al., 2023). The inability of mNGS to distinguish viable from nonviable organisms further complicates interpretation, especially in patients who received empirical antibiotics before specimen collection, as residual DNA from suppressed organisms may persist despite loss of viability (Gu et al., 2019; Hao et al., 2024; Kalbitz et al., 2024).

The discordant results observed between mNGS and culture in this study highlight the complementary nature of the two diagnostic modalities. Cases that were culture-positive but mNGS-negative may reflect low sequencing depth, DNA degradation, or organisms with limited genomic representation in reference databases (Zheng et al., 2023; Kalbitz et al., 2024; Lei et al., 2025). Conversely, cases that were mNGS-positive but culture-negative likely reflect the known limitations of anaerobic culture, including oxygen sensitivity during transport and the difficulty of cultivating fastidious organisms. Dual-negative cases may be attributable to low bacterial load, inadequate sampling, or suppression of viable organisms by prior antibiotics. These patterns underscore the need for a composite clinical reference standard—integrating clinical presentation, imaging, surgical findings, and organismal abundance—when interpreting microbiological results in DNSI.

The biological complexity of polymicrobial infections in DNSI warrants further emphasis. Synergistic interactions between anaerobic and aerobic organisms, biofilm formation, tissue hypoxia, and host immune dysregulation all contribute to pathogen behavior and disease severity. These mechanisms are particularly relevant in patients with metabolic dysfunction, such as diabetes mellitus or overweight status, conditions associated with impaired neutrophil function, chronic inflammation, and altered oral microbiota.In this study, Streptococcus constellatus and Prevotella melaninogenica were frequently detected in diabetic and overweight patients, consistent with prior reports linking these organisms to polymicrobial head and neck infections in metabolically compromised hosts (Negrini et al., 2021; Schamarek et al., 2023; Kong et al., 2024). However, subgroup analyses were limited by small sample sizes and should be interpreted cautiously.

Although antimicrobial regimens were adjusted in selected patients after mNGS reporting, improvements in inflammatory markers likely reflected the combined effects of surgical drainage, empirical broad-spectrum antibiotics, and supportive care, and cannot be ascribed specifically to mNGS-informed antimicrobial changes. Surgical source control remains the cornerstone of DNSI management, and molecular diagnostics cannot replace the need for timely operative intervention. Furthermore, the retrospective design of this study precludes establishing causal relationships between mNGS-informed antimicrobial adjustments and clinical outcomes. Statements implying therapeutic benefit have therefore been tempered to reflect observational associations only.

The potential for overdiagnosis and overtreatment represents an important consideration when interpreting mNGS results. Ultra-sensitive molecular detection methods may identify low-abundance organisms, commensals, or contaminants that do not contribute to the infectious process, and unnecessary antimicrobial escalation may occur if such findings are overinterpreted. Conversely, mNGS may provide clinically useful information in selected cases, particularly when culture is negative or when fastidious or anaerobic organisms are suspected. The challenge lies in integrating sequencing data with clinical context to avoid both under- and over-treatment.

Future prospective multicenter studies integrating standardized sampling procedures, contamination control strategies, host inflammatory biomarkers, and quantitative sequencing thresholds will be necessary to establish evidence-based interpretive frameworks for mNGS in polymicrobial deep neck infections (Kalbitz et al., 2024; Zhang et al., 2024).

## Conclusion

In this retrospective observational study of patients with deep neck space infections who underwent surgical drainage, metagenomic next-generation sequencing (mNGS) provided a broader detection spectrum and a substantially shorter reporting time than conventional culture, particularly for anaerobic and fastidious organisms that are difficult to recover under routine laboratory conditions. However, because mNGS detects microbial DNA irrespective of organism viability and frequently identifies oral commensals or low-abundance taxa in polymicrobial environments, its interpretation requires careful integration with clinical presentation, anatomical involvement, inflammatory markers, and intraoperative findings. The discordant patterns observed between mNGS and culture underscore the complementary nature of the two diagnostic modalities and highlight the need for a composite clinical reference standard when evaluating microbial relevance in deep neck infections. Although antimicrobial regimens were adjusted in selected patients after mNGS reporting, improvements in inflammatory markers likely reflected the combined effects of surgical drainage, empirical therapy, and supportive care, and cannot be attributed specifically to mNGS-associated antimicrobial changes within the limitations of this retrospective study.Taken together, these findings suggest that mNGS may serve as a useful adjunct to conventional diagnostics in selected cases of deep neck space infection, particularly when anaerobic or fastidious organisms are suspected or when culture results are inconclusive. Future prospective controlled studies with standardized interpretive frameworks are needed to clarify the incremental clinical value of mNGS and to define its appropriate role within the broader diagnostic and therapeutic strategy for deep neck space infections.

## Data Availability

The raw metagenomic sequencing data generated in this study have been deposited in the NCBI Sequence Read Archive (SRA) under BioProject accession number PRJNA1476743 and SRA Study accession number SRP708436. The deposited sequencing data can be accessed through the NCBI BioProject database at: https://www.ncbi.nlm.nih.gov/bioproject/PRJNA1476743. Raw sequencing data are available for 27 of the 32 study participants and have been deposited in the repository under SRA accession numbers SRR39090568–SRR39090594. Raw sequencing data for five patients (Patients 2, 3, 5, 11, and 31) are unavailable because the original sequencing files were irretrievably lost following a laboratory information system upgrade after completion of the analyses. The corresponding mNGS analysis reports for these patients are provided as **Supplementary File S1**. All other data supporting the findings of this study are included within the article and its **Supplementary Material**.
