# Supplementary material for: Diagnostic value of metagenomic next-generation sequencing in deep neck space infections: a retrospective study of 32 patients
Source: Front Cell Infect Microbiol. 2026 Jul 17;16:1874210. doi: 10.3389/fcimb.2026.1874210 (PMC13423987; doi:10.3389/fcimb.2026.1874210)
Supplement: Supplementary file 1 [file DataSheet1.pdf]

Patients 2

Detailed list of test results

检测结果详细列表

细菌筛查结果									
类型	属			种					
	中文名	拉丁文名	序列数	中文名	拉丁文名	RPM	覆盖度(%)	相对丰度(%)	置信度
G+	微单胞菌属	*Parvimonas*	4,694	微小微单胞菌	*Parvimonas micra*	388.85	19.37	30.86	高

病毒筛查结果
未检出

真菌筛查结果
未检出

寄生虫筛查结果
未检出

特殊病原体列表（结核分枝杆菌复合群/非结核分枝杆菌/支原体/衣原体/立克次体/螺旋体）
未检出

耐药基因筛查结果
未检出

Patients 3

Detailed list of test results

检测结果详细列表

细菌筛查结果
未检出
**病毒筛查结果**
未检出
**真菌筛查结果**
未检出
**寄生虫筛查结果**
未检出
**特殊病原体列表（结核分枝杆菌复合群/非结核分枝杆菌/支原体/衣原体/立克次体/螺旋体）**
未检出
**耐药基因筛查结果**
未检出

Patients 5

Detailed list of test results
检测结果详细列表

<table>
<tr><th colspan="10">细菌筛查结果</th></tr>
<tr><th rowspan="2">类型</th><th colspan="3">属</th><th colspan="6">种</th></tr>
<tr><th>中文名</th><th>拉丁文名</th><th>序列数</th><th>中文名</th><th>拉丁文名</th><th>RPM</th><th>覆盖度(%)</th><th>相对丰度(%)</th><th>置信度</th></tr>
<tr><td>G+</td><td>链球菌属</td><td>Streptococcus</td><td>506,105</td><td>化脓链球菌</td><td>Streptococcus pyogenes</td><td>32613.27</td><td>99.17</td><td>99.77</td><td>高</td></tr>
<tr><th colspan="10">病毒筛查结果</th></tr>
<tr><td colspan="10">未检出</td></tr>
<tr><th colspan="10">真菌筛查结果</th></tr>
<tr><td colspan="10">未检出</td></tr>
<tr><th colspan="10">寄生虫筛查结果</th></tr>
<tr><td colspan="10">未检出</td></tr>
<tr><th colspan="10">特殊病原体列表（结核分枝杆菌复合群/非结核分枝杆菌/支原体/衣原体/立克次体/螺旋体）</th></tr>
<tr><td colspan="10">未检出</td></tr>
<tr><th colspan="10">耐药基因筛查结果</th></tr>
<tr><td colspan="10">未检出</td></tr>
</table>

Patients 11

Detailed list of test results

检测结果详细列表

细菌筛查结果								
未检出								
**病毒筛查结果**								
未检出								
**真菌筛查结果**								
属			种					
中文名	拉丁文名	序列数	中文名	拉丁文名	RPM	覆盖度(%)	相对丰度(%)	置信度
糖酵母属	*Saccharomyces*	211	酿酒酵母	*Saccharomyces cerevisiae*	1.3	0.14	2.96	中
**寄生虫筛查结果**								
未检出								
**特殊病原体列表（结核分枝杆菌复合群/非结核分枝杆菌/支原体/衣原体/立克次体/螺旋体）**								
未检出								
**耐药基因筛查结果**								
未检出								

Patients 31

Detailed list of test results
检测结果详细列表

细菌筛查结果									
类型	属			种					
	中文名	拉丁文名	序列数	中文名	拉丁文名	RPM	覆盖度(%)	相对丰度(%)	置信度
G+	微单胞菌属	*Parvimonas*	283,129	微小微单胞菌	*Parvimonas micra*	12165.05	71.04	6.19	高
G-	普雷沃菌属	*Prevotella*	1,865,941	口腔普雷沃菌	*Prevotella oris*	75623.68	70.81	38.50	高
G+	链球菌属	*Streptococcus*	1,182,364	星座链球菌	*Streptococcus constellatus*	25047.43	83.58	12.75	高

病毒筛查结果
未检出

真菌筛查结果
未检出

寄生虫筛查结果
未检出

特殊病原体列表（结核分枝杆菌复合群/非结核分枝杆菌/支原体/衣原体/立克次体/螺旋体）
未检出

耐药基因筛查结果
未检出
